# Adapting blockchain’s proof-of-work mechanism for multiple traveling salesmen problem optimization

**DOI:** 10.1038/s41598-023-41536-0

**Published:** 2023-09-06

**Authors:** Nareman Sabry, Bahaa Shabana, Mohamed Handosa, M. Z. Rashad

**Affiliations:** 1 Department of Computer Science, Faculty of Computers and Information, Mansoura University, Mansoura, 35516 Egypt; 2 Computer Science Department, Misr Higher Institute for Commerce and Computers, Mansoura, Egypt

**Keywords:** Computational science, Computer science, Software

## Abstract

The blockchain network uses a Proof-of-Work (PoW) mechanism to validate transactions and keep the blockchain growth safe against tampering, but it is hugely energy-consuming with no benefit to the peer-to-peer network participants. In this paper, we proposed a blockchain network for distributing products to different locations based on the use of the Proof of Useful Work mechanism, in which miners use computing resources to optimize the traveling salesman problem (TSP) as an alternative to solving mathematical problems that represent the basis of the traditional PoW mechanism to get a new block. According to this proposed blockchain, it not only receives and securely stores the distribution locations but also improves the paths for salesmen when traveling between different locations during the transportation process. This strategy aims to take advantage of the miners’ efforts to minimize the traveled distance by applying the clustering technique and computing the shortest path by Guided Local Search (GLS) for each cluster at the same time. According to the tested results on TSP-LIB instances, the used strategy works efficiently with an average of 0.08 compared to the rest of the meta-heuristics, and the proposed architecture reduced total distances with an average of 0.025%. In addition, the block generation time in the blockchain decreased by 11.11% compared to other works.

## Introduction

**Figure 1:**
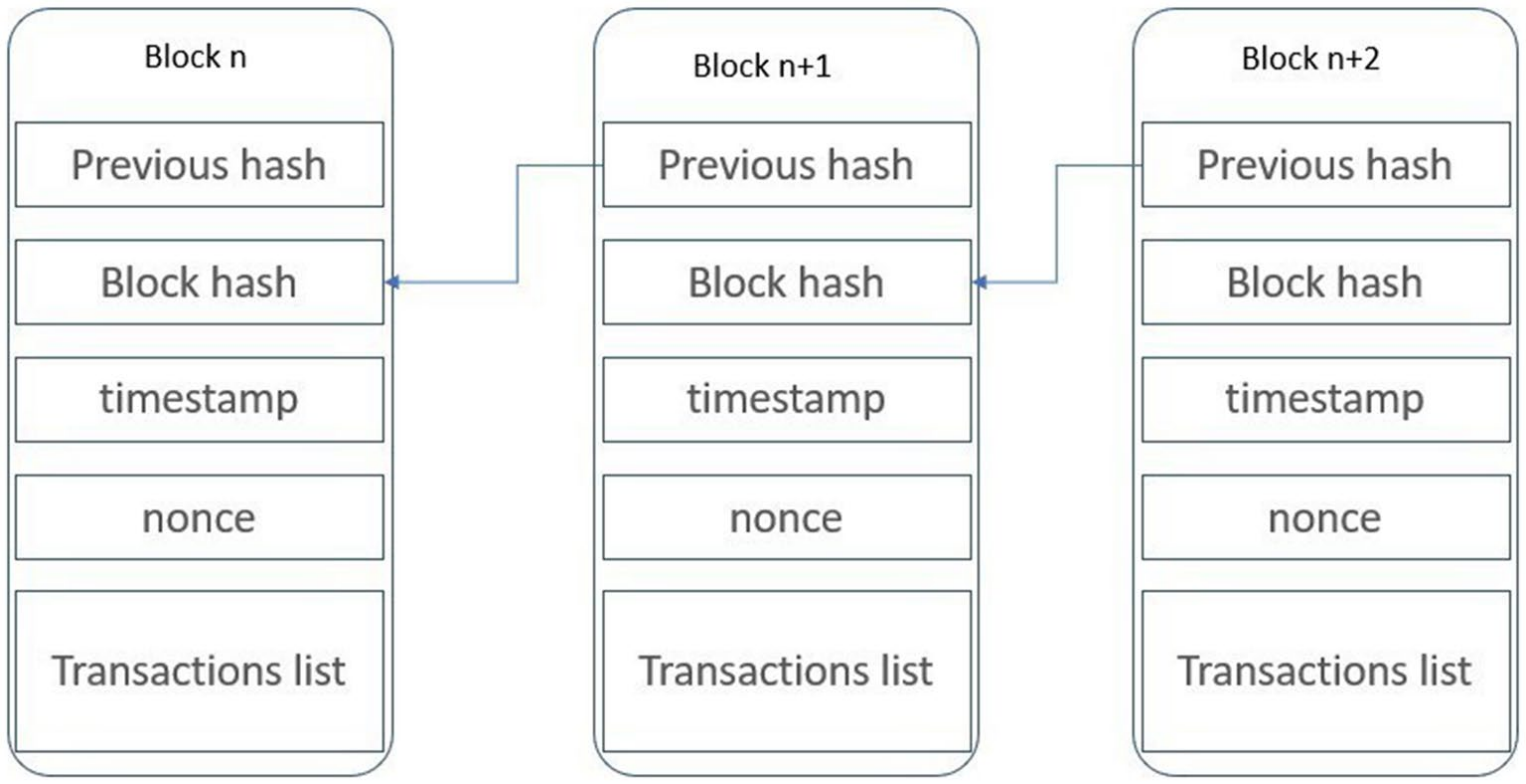
Organizing and linking blocks in the chain.

Blockchain is a decentralized and persistent ledger that facilitates transaction recording and resource management in peer-to-peer networks. Initially introduced through Bitcoin by Nakamoto^1^, blockchain has gained widespread adoption in various industrial applications due to its unique characteristics, including transparency, durability, and security, which traditional databases lack^2^. It operates as a chain of interconnected and chronologically organized blocks, as depicted in Fig. 1. Each block’s data is encrypted using algorithms like sha256^3^, ensuring its integrity and including the previous block hash, thus preserving immutability. Additionally, each block comprises a list of validated transactions, a timestamp denoting block creation time, and a nonce value, a unique random number assigned to the block^4^. A consensus mechanism protocol is followed by all network participants when a new block is added to the blockchain, allowing nodes to confirm the validity and permission of the new block’s transactions. The most prominent consensus mechanism is Proof of Work (PoW), wherein miners compete to solve a challenging mathematical puzzle to find a valid nonce for the block, and successful miners are rewarded for their computational efforts. However, this mechanism incurs significant energy consumption without offering additional benefits to network participants^5^. Studies show that Bitcoin mining alone consumes an estimated 0.1 to 10 GW of energy, exceeding the average electricity consumption of Ireland, which stands at 3 GW^6^. Nonetheless, PoW remains a practical and indispensable consensus mechanism for effective transaction validation in the blockchain ecosystem.

The Traveling Salesman Problem (TSP) and the multiple Traveling Salesman Problem (mTSP) address finding the shortest route challenge for a salesman to visit multiple locations and return to the starting point. Recently, these computational problems have wide-ranging applications in optimizing logistics and transportation. While various algorithms exist to solve these problems, finding the optimal solution requires dedicated effort and exploration.

Therefore, it is better to utilize wasted mining energy by converting proof of work into proof of useful work by solving optimization problems like the TSP. Accordingly, this paper aims to redirect computational power in blockchain validation towards meaningful challenges, benefiting participants and enhancing blockchain capabilities. This approach transforms a notable weakness into a valuable competitive advantage.

The main contributions of this study are summarized as follows:

- We employed a new PoUW that generates meaningful outputs for a road transportation blockchain, demonstrating the utilization of mTSP optimization as the miner’s task, while keeping in mind the system’s security requirements as well as the rewards offered to miners.
- The proposed technique describes an optimization problem with the objective of reducing the overall transportation distance between identical origin and destination locations in the mTSP.
- The proposed consensus process allows clustering locations to reduce costs and then utilizing GLS as a specific cascade process for miners in the PoUW to choose the optimal solution to create a valid block, which leads to a reduction in the block generation time.

The rest of the paper is organized as follows: in “Literature review” section, some related works for leveraging the blockchain’s POW power in other areas will be discussed. In “System model” section, the system model is listed including the System nodes, PoUW consensus mechanism, Threat model, and Rewards distribution mechanism. The security analysis is presented in “security analysis” section. In “Implementation details” section, the implementation details are explained. In “Experimental results” section, the results obtained from the implementation will be shown. Discussion is conducted in “Discussion” section. Finally; the conclusion and future work will be presented in “Conclusions and future work” section.

## Literature review

The major drawback of PoW is its high energy consumption due to the extensive computing power needed to solve cryptographic puzzles. In response to this issue, Proof of Stack (PoS)^7^ has been proposed as an alternative. PoS dynamically adjusts the puzzle difficulty for each node based on their token holdings, favoring nodes with more token age consumed (TAC) to validate blocks. Additionally, other consensus mechanisms like Proof of Luck^8^ and Robust Proof of Stake^9^ offer energy-efficient and sustainable solutions for blockchain networks. Alongside these alternatives, Block-DEF^10^ provides secure and tamper-proof evidence management with its interconnected service, blockchain, and network layers. Despite these options, PoW remains widely accepted, particularly in permissionless blockchain architectures.

Several researchers have devoted their efforts to harnessing the energy of PoUW and maximizing its potential to tackle critical challenges. Syafruddin et al.^11^ utilized the Traveling Salesman Problem (TSP) as an optimization task within the POW framework. By employing the Particle Swarm Optimization metaheuristic in the SOLVER class, they encoded solutions and evaluated fitness, enabling miners to discover the most cost-effective path and significantly enhancing blockchain strategies. Mittal et al.^12^ introduced a PoUW known as Proof of Deep Learning with Hyper-Parameters Optimization. This approach capitalized on the surplus energy from hashing. Through competition among miners and the utilization of Bayesian optimization techniques coupled with the MNIST dataset, their models achieved high-performance levels. To further exploit the power of POW for Deep Learning, Chenli, Changhao, et al.^13^ proposed DL-chain. This innovative method employed the Raft algorithm to select a publisher responsible for broadcasting training tasks. Notably, DL-chain bolstered security measures, requiring substantial power for potential attacks. Liu et al.^14^ presented Proof of Learning (PoLe), which repurposed computing power to facilitate neural network training, combining a secure mapping layer (SML) served as a deterrent against theft and fostered collaboration among data nodes while fostering competition among consensus nodes. In the domain of transportation request aggregation, Haouari et al.^15^ leveraged POW to solve the challenge by employing a concave cost function. Their approach, formulated as a mixed-integer nonlinear programming problem, was successfully resolved using the branch-and-cut technique. The outcome was a substantial 35% reduction in transportation costs. N Lasla, et al.^16^ proposed Green-PoW, an energy-efficient consensus algorithm for PoW to reduce energy consumption by alternating mining rounds and selecting a subset of miners for exclusive participation in the second round. This technique preserved energy consumption by up to 50%, improved security by reducing fork occurrences, and reduced mining centralization minimizing overall energy use during mining.

In summary, the listed studies are limited in effectively addressing Proof-of-Work power consumption and exploitation in different fields, but they increased the complexity of the consensus process for miners, which can lead to an increase in block generation time. Therefore, the proposed PoUW avoided these shortcomings by reducing the block generation time because the consensus nodes follow a sequential technique every time they optimize mTSP and search for the valid nonce to create the block, which led to a significant reduction in energy due to the reduction of the block generation time, taking security standards into account.

## System model

The proposed system is a decentralized system consisting of nodes responsible for initializing optimization tasks and other nodes to solve these tasks and earn rewards through secure communication. The system components are depicted as follows:

### System nodes

Our proposed decentralized blockchain system consists of multiple nodes distributed across the network, where each node acts as a data node or a consensus node (miner). Data nodes store mTSP instances and solutions with location (coordinates of cities) and path details (sequences of cities in the solution). Stakeholders, like businesses and organizations, provide optimization tasks and mTSP instances, attaching rewards or fees within the network. Meanwhile, consensus nodes actively contribute computational resources, competing for the offered tasks and receiving rewards in return. This decentralized architecture ensures efficiency, transparency, and effectiveness in optimizing the mTSP problem.

### PoUW (proof-of-useful-work) consensus mechanism

The mining process concerns the preface of computational complexity through the PoUW consensus mechanism. Miners actively try to extract nonce n that, upon hashing with the block header H, results in a hash value H′ lower than the target value T. This process is represented in Eq. (1):

1 $$ H^{\prime } = Hash\left( {H,n} \right) < T $$

where T is dynamically adjusted to regulate mining difficulty and ensure a steady block generation rate.

**Figure 2:**
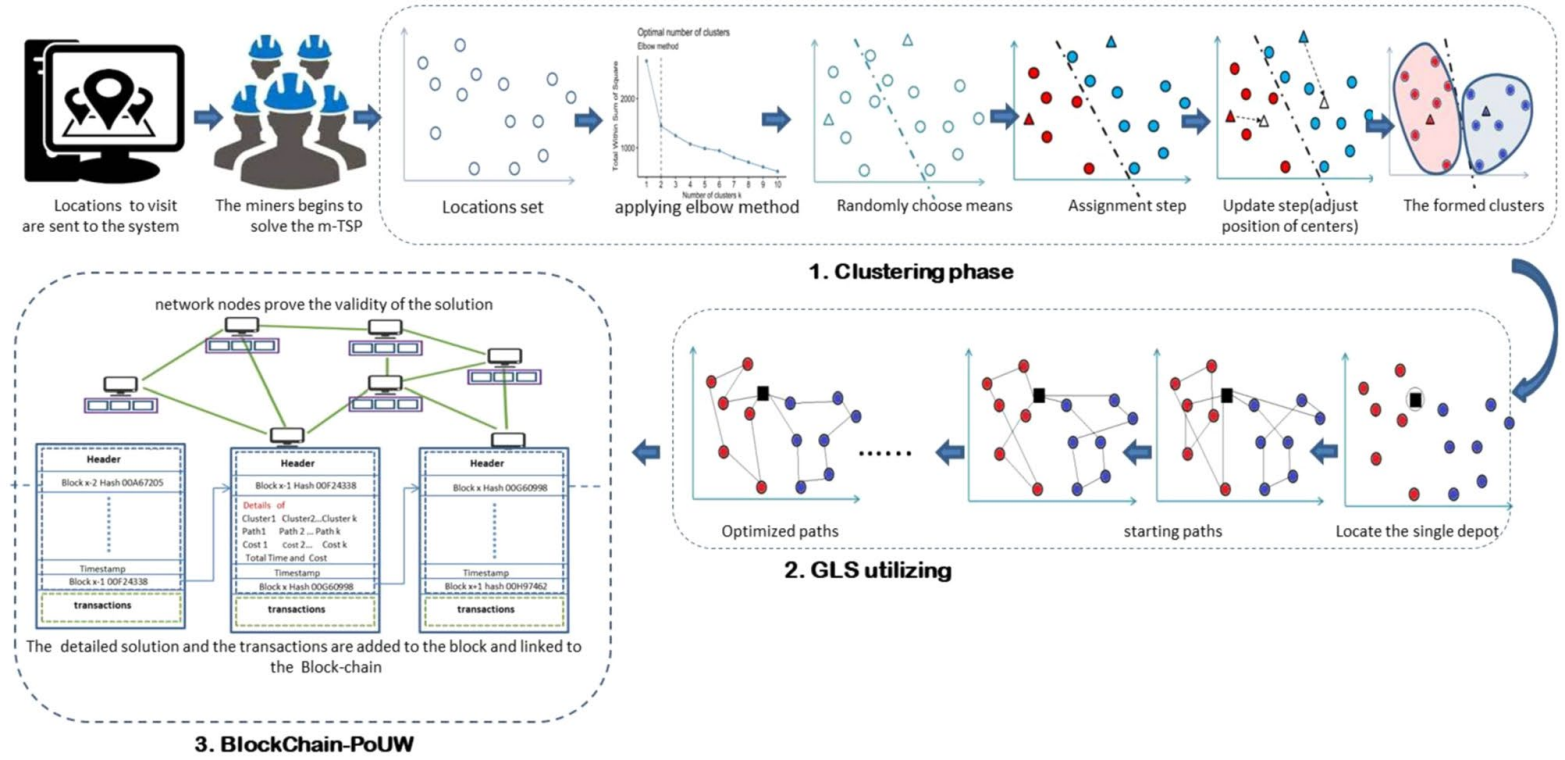
Miner’s challenge phases in the proposed architecture.

The proposed miner’s challenge, which utilizes the blockchain’s proof of work to solve the problem of multiple salesmen, is divided into three basic phases, as shown in Fig. 2, and each phase is defined as follows:

- *Clustering phase* Refers to the partition of locations set into a number of clusters by repeating the two steps of assignment and updating.
- *GLS utilization* Involves applying this algorithm to the locations of each formed cluster to optimize each cluster’s path as much as possible.
- *Blockchain system (PoUW)* Receives and stores optimized paths, then broadcasts them to salesmen.

The proposed PoUW is explained as:

#### Clustering phase

**Figure 3:**
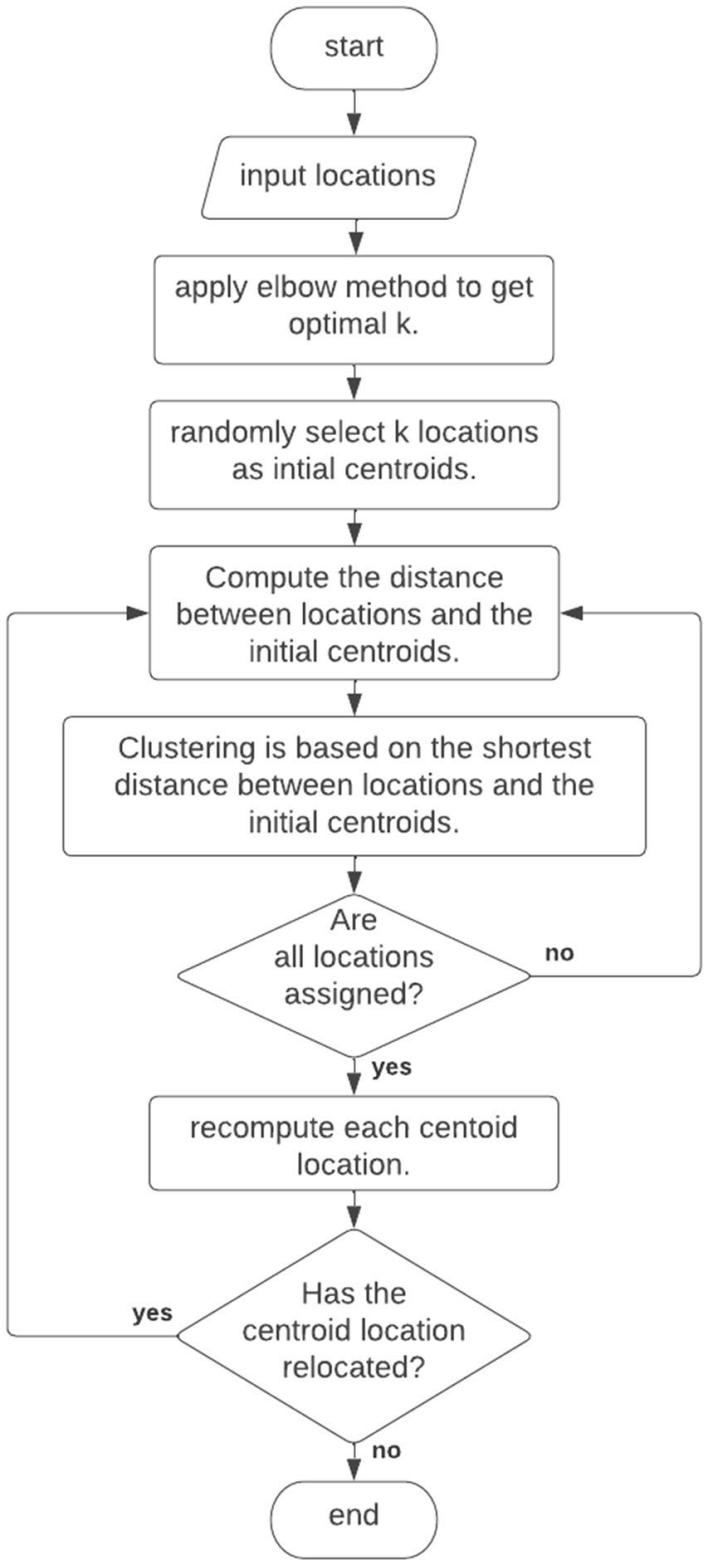
Locations division by K-means clustering algorithm.

The K-means algorithm is utilized in the first phase to cluster a set of locations^17^, denoted as, int K clusters, represented as X = (x1,…,xn}, into K clusters, represented as {C1,…,Ck}. It starts by randomly selecting k centroid locations as initial centers, evaluating distances between these centers and locations, assigning each location to the nearest centroid’s cluster, recalculating centroids for each cluster, and iterating until convergence. Figure 3 illustrates the flowchart of the clustering process for input locations by the k-means.

The clustering phase consists of repeating the assignment and updating steps as follows:

*Step 1* Assignment stage

- The miners use the elbow method to find the optimum k number during a given range to increase the efficiency of splitting locations^18^, and set k-means points randomly as the center of each cluster *µ*_1_,*µ*_2_,…,*µ*_*k*_.
- The distance between each location and the center is measured by the Euclidean distance Eq. (2):
  2 $$ d\left( {x,\mu_{i} } \right) = \sqrt {\left( {x_{1} - x_{2} } \right)^{2} + \left( {y_{1} - y_{2} } \right)^{2} } $$

Where *d* is the distance between each location *x* and each center/mean *µ*_*i*_, x has coordinates *x*_1_*, y*_1_, and *µ*_*i*_ has coordinates *x*_2_*, y*_2_.

Assign the location to the nearest center, as in Eq. (3):

3 $$C_{i}=\left\{x:\left\|x-\mu_{i}\right\|^{2} \leq\left\|x-\mu_{j}\right\|^{2} \forall j, 1 \leq j \leq k\right\}$$

where* C*_*i*_ is cluster i, *x* is the assigned location*, µ*_*i*_ is the center of cluster i, *µ*_*j*_ represents the center of cluster j, and the number of clusters varies from 1 to k.

*Step 2* Update stage

Modify the means for the locations assigned to each cluster, as in Eq. (4):

4 $$  \mu_{i} = \frac{1}{{c_{i} }}\mathop \sum \limits_{{x_{i} \in C_{i} }} x_{i} $$

where *c*_*i*_ is the number of locations in the cluster *C*_*i*_.

Repeat the assignment and update steps until the cluster centers haven’t changed more

**Figure 4:**
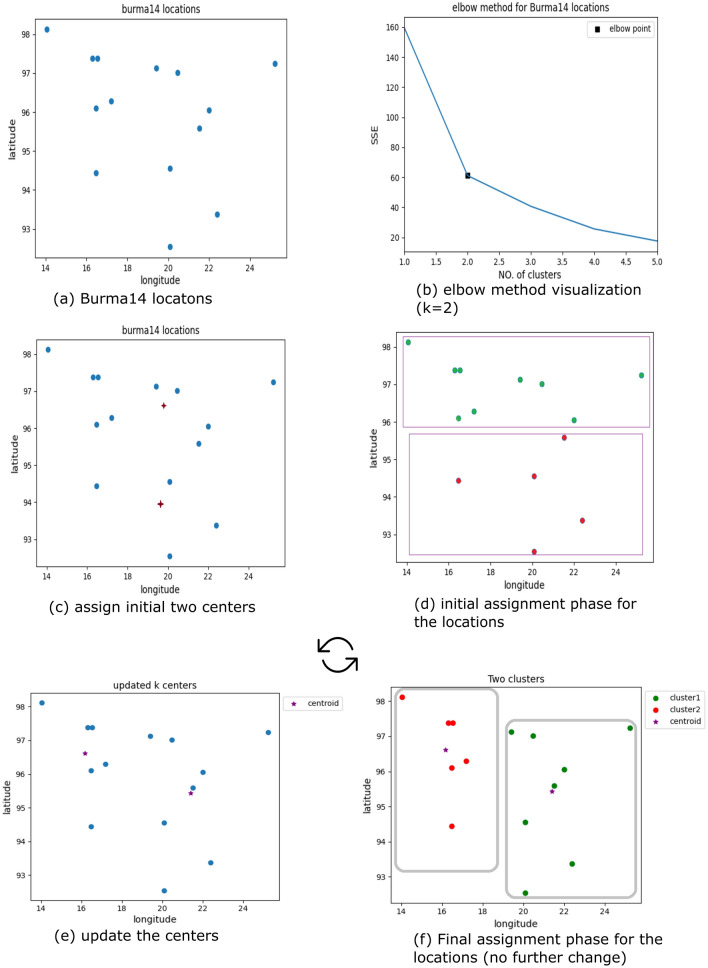
Applying the k-means algorithm on the Burma14 locations.

Figure 4 shows the output of the clustering steps of the k-means algorithm applied to the Burma14 data set, which includes 14 geographical coordinates for the cities in Burma, and the final distribution of these locations is shown in Table 1.

**Table 1:** Distribution of the TSP-LIB instance (burma14) locations.

Node	Longitude	Latitude	Cluster
1	16.47	96.1	1
2	16.47	94.44	1
3	20.09	92.54	2
4	22.39	93.37	2
5	25.23	97.24	2
6	22	96.05	2
7	20.47	97.02	2
8	17.2	96.29	1
9	16.3	97.38	1
10	14.05	98.12	1
11	16.53	97.38	1
12	21.52	95.59	2
13	19.41	97.13	2
14	20.09	94.55	2

#### GLS utilization

Guided Local Search (GLS) is a powerful metaheuristic optimization method, recognized for its ability to escape local optima and find better solutions by using a penalty-based strategy^19^. In the context of finding the best path for a set of locations, GLS is utilized to improve the solution by penalizing certain features (edges) and adjusting the cost function iteratively. The indicator function indicates or predicts whether the feature is in the solution or not, as in Eq. (5):

Assuming *s* is a given solution/path, *i* is a feature (The edge between every two locations).

5 $$ I_{i} \left( s \right) = \left\{ {\begin{array}{*{20}l} 1 \hfill &\, {{\text{if\, i}} \in {\text{s}},} \hfill \\ 0 \hfill &\, {{\text{otherwise}}.} \hfill \\ \end{array} } \right. $$

where *s* is the given solution and *i* is the feature (edge).

The steps involved in using GLS to get the best path for locations are as follows:

- Determine the depot point for the salesman’s departure and return, then add it to each cluster.
- Start with an initial solution/path *s*_∗_, which is a local optimum.
- Evaluate the utility of each feature in the path using Eq. (6):
  6 $$ util_{i} \left( {s_{*} } \right) = I_{i} \left( {s_{*} } \right) \cdot \frac{{c_{i} }}{{1 + p_{i} }} $$

Where *c*_*i*_ represents the cost of a feature *i* in solution *s*_∗_.

- Increase the penalties for the features with the highest utility by 1, guiding the search away from locally optimal solutions.
- Repeat the search process from the same local optimum *s*_∗_, applying the enhanced augmented function h(s) to the original objective function g(s), which calculates the minimum cost Hamiltonian cycle TSP, ensuring each node is visited exactly once and returns to the starting point^20^, as in Eq. (7), (8)^21^:
  7 $$  g\left( s \right) = {\text{min}}\mathop \sum \limits_{i,j \in s}^{N} d_{ij} $$

Where *d*_*i j*_ represents the distance from node *i* to node *j* in path *s*.

8 $$  {\text{h}}\left( {\text{s}} \right) = {\text{g}}\left( {\text{s}} \right) + \lambda \mathop \sum \limits_{{{\text{i}} \in {\text{F}}}} {\text{p}}_{{\text{i}}} {\text{*I}}_{{\text{i}}} \left( {\text{s}} \right) $$

where *λ* is a penalty-scaling factor that influences the search behavior to explore similar (low *λ*) or distinct (high *λ* ) solutions. *F* represents the set of features (edges), and *p*_*i*_ represents the penalty of each feature (initially set to 0).

**Figure 5:**
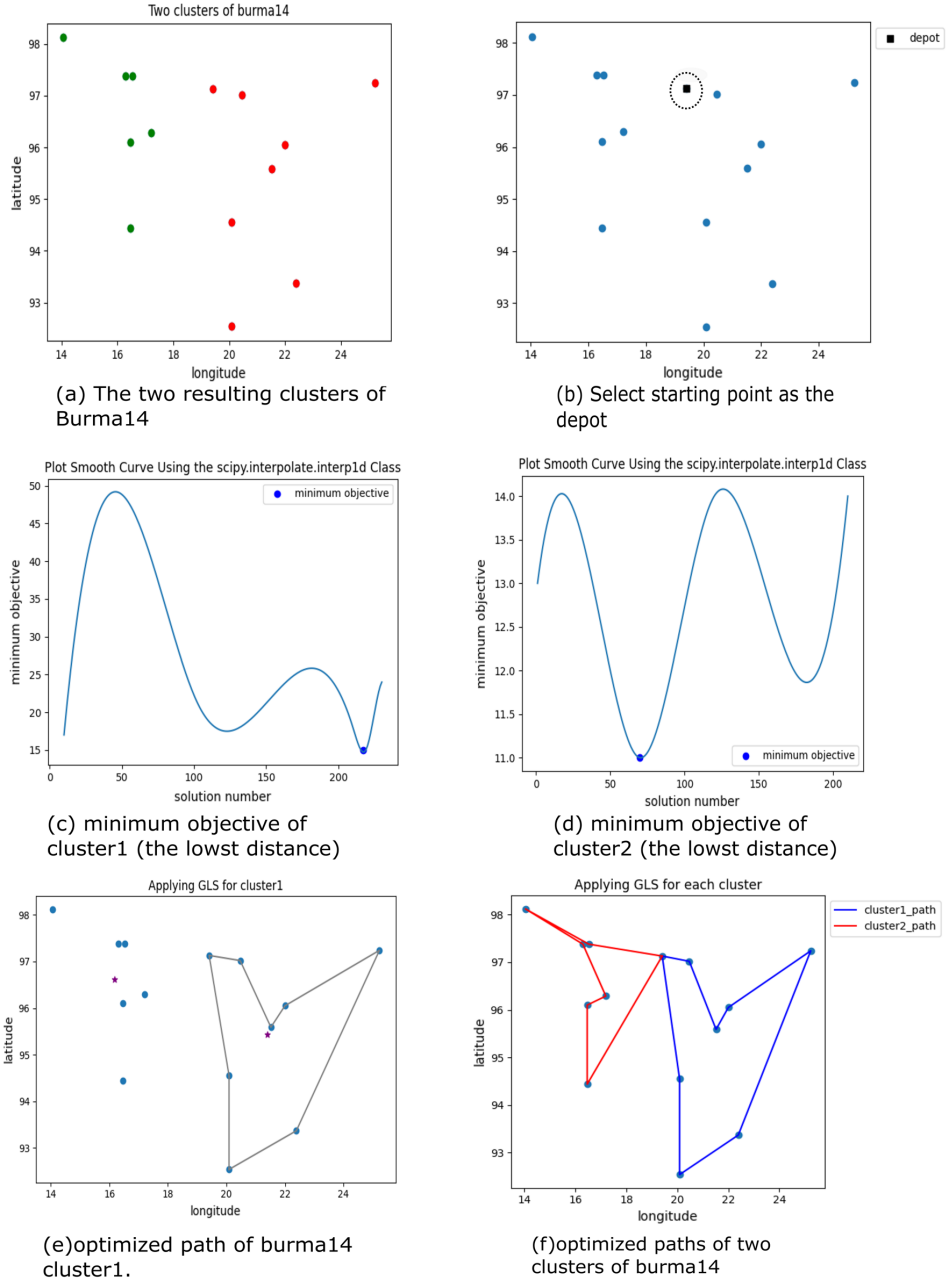
GLS Utilization to find the shortest possible path for each cluster.

Guided Local Search efficiently explores the solution space to find the best path of locations by iteratively adjusting the cost function and applying penalties. The algorithm continuously tests solutions, escaping from the local optimum until reaching the lowest possible objective. Figure 5 illustrates the resulting paths for the complete m-TSP solution, achieving shorter total distances traveled by each salesman. Table 2 presents the sequence of locations and their respective path costs.

**Table 2:** Sequence of paths and cost for each cluster of burma14.

Clusters	Path	Cost
1	(19.41, 97.13), (20.47, 97.02), (21.52, 95.59), (22., 96.05), (25.23, 97.24), (22.39, 93.37), (20.09, 92.54), (20.09, 94.55), (19.41, 97.13)	15
2	(19.41, 97.13), (16.47, 94.44), (16.47, 96.1), (17.2, 96.29), (16.3, 97.38), (14.05, 98.12), (16.53, 97.38), (19.41, 97.13)	11

#### Blockchain system (PoUW)

**Figure 6:**
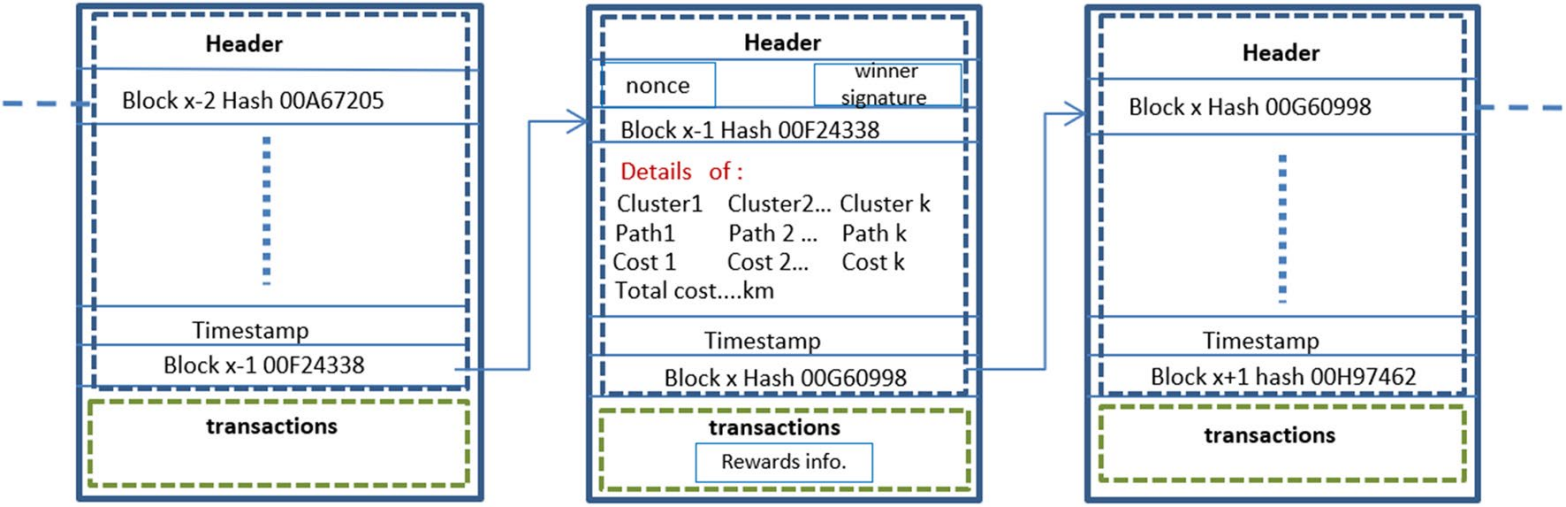
Data structure in linked blocks of the proposed Blockchain.

Miners create valid blocks with solutions and share them with all nodes. Nodes verify block integrity, miner identity correctness, and mTSP authenticity for consensus. Upon unanimous agreement, a valid block is added to the blockchain. The block’s data structure includes a header containing the block hash, the previous block hash, timestamp, nonce, and encrypted signature of the winner miner as metadata, the obtained optimized mTSP solution, and transaction data with winner miner rewards. Each block is linked to the hash of the previous block to prevent data tampering, as shown in Fig. 6 The new block is added to the blockchain simultaneously by all nodes, ensuring a secure and reliable blockchain for optimizing the mTSP.

### Threat model

The threat model aims to identify potential attack risks and vulnerabilities in POUW that may pose a risk to system security.

Threat actors:

- *Malicious miners* These actors take passive actions to disrupt the functionality of the blockchain network, engaging in actions such as producing invalid or fraudulent blocks, launching double-spending attacks, or rejecting valid blocks to slow down the consensus process.
- *External attackers* Refer to entities or people outside the PoUW system attempting to exploit vulnerabilities with the aim of unauthorized access to the PoUW system’s components or communication channels to steal data or disrupt the blockchain network. Table 6 shows some examples of attacks on the system, with their classification as external or internal. Table 3 shows some examples of attacks on the system, with their classification as Malicious Miners or External Attackers
- *Colluding consensus nodes* This type of attacker represents interested consensus nodes who try to collude and cooperate for illegal earnings, potentially forming mining pools to dominate mining power and control block creation.

**Table 3:** Comparison of the threat actors and some of the attack scenarios and corresponding examples.

Threat actor	Threat scenario	Example
Malicious miners	51% Attack^22^	Malicious miners set controls over 50% of the blockchain's computational power, threatening transaction integrity
External attackers	Double-spending^23^	In a decentralized mTSP environment, external attackers try to utilize the same cryptocurrency multiple times
	Denial of service (DoS) and distributed DoS^24^	Attackers overload the system with requests, preventing genuine users from accessing it
	Sybil attacks^25^	Attackers create multiple Sybil nodes or identities to control the network and disrupt consensus
	Eclipse attacks^26^	Attackers separate a node from the network in order to influence its routing choices or deny it access to certain information

Threat indicators:

- *Uncommon block patterns* Rapid growth in the number of fraudulent blocks within the blockchain indicates the possibility of malicious miners manipulating the blockchain.
- *Anomalous mining procedures* Unusual distribution of mining authority, indicating potential collusion attempts or malicious intent.
- *Irregular communication traffic* Strange network communication flows are indicators of Distributed Denial of Service (DDoS) attacks or efforts to disrupt the network’s functionality.

Mitigation procedures:

Some security standards are adapted to counter these threats, as follows:

- Use cryptography algorithms for hashing data and create digital signatures for miners, to preserve the integrity of data-optimized solutions and their owner identity.
- Establish a secure nonce management mechanism to prevent miners from faking or manipulating workloads by applying specific criteria for nonce generation to ensure the PoUW’s integrity.
- Real-time monitoring and alerting processes are implemented to notice and mitigate potential threats by transmitting email notifications to system members of abnormal or suspicious activities.
- Offer advancing Support for the miners to address their security issues as questions by accessing security specialists or a specialized support team.

Implementing all of these comprehensive mitigation measures, the proposed PoUW secures from possible threats.

### Rewards distribution mechanism

The rewards distribution mechanism in our proposed PoUW improves motivation and transparency among miners. It considers their computational efforts, valid block mining, and overall network performance to ensure fitting and proportional reward allocation, as calculated in the Eq. (9):

9 $$ R_{i} = \frac{{W_{i} \times B}}{T} $$

where *R*_*i*_ is the reward earned by miner *i*, *W*_*i*_ denotes the computational effort performed by miner *i*, *B* is the block reward for mining a new block, *T* denotes the total computational effort accomplished by all miners in the network.

The optimization tasks payment could be obtained by various stakeholders, such as individuals, businesses, or organizations, who require solutions to mTSP instances. They initiate transactions within the blockchain network and attach a reward or fee for the miners’ efforts.

## Security analysis

This section outlines how the proposed model addresses possible security threats as follows:

### Data integrity and chain revision

In our PoUW, data integrity is a priority, achieved through SHA-256 hashing, which generates distinct fixed-size hash values for each block, transaction and PoUW consensus. We prevent tampering by generating unique hash values for each block and employing computational complexity to counter chain revision attacks. Each block includes the previous block hash, providing the chain’s integrity. This provides a robust platform for real-world optimization challenges.

### Model theft

In this threat, consensus nodes try to claim ownership of optimized mTSP solutions by broadcasting them to the blockchain network. To prevent this, we implemented Secure Mining Logic (SML), which acts as a unique digital identity or fingerprint for each optimized solution produced by miners.

The implementation of SML involves the following steps:

- *Hashing solution information* Calculate a hash value for the optimized mTSP solution using SHA-256 cryptographic hashing algorithms. This generates a fixed-size hash representing the solution’s information.
- *Encryption of the previous hashed solution* The generated hash value of the solution was encrypted using the Advanced Encryption Standard (AES) encryption algorithm^27^. This extra layer of encryption provides the confidentiality and protection of the mTSP solution.
- *Creating verification keys* Public–Private key pairs are generated for each consensus node (miner) in the blockchain network for security. The public key verifies the authenticity of the miner’s signature, and the private key allows miners to generate digital signatures. These keys to ensuring the integrity and ownership verification of the optimized mTSP solutions.
- *Miner signature generation* Consensus nodes use their private keys to generate digital signatures, which act as cryptographic proofs, proving ownership of the optimized mTSP solution and establishing a safe link between it and the specified node, which improves the overall security of the blockchain-based mTSP optimization process.

### Addressing consensus node collusion

We addressed the issue of collusion among consensus nodes, also known as the 51% attack, which occurs when extending blockchains with the PoUW node consensus and a set of nodes forms a mining pool and generates the same new block. There are two reasons why collusion becomes hard in our proposed system: (1) PoUW is resource-intensive: The proposed PoUW consensus is designed for the mTSP optimization problem, requiring miners to use K-means clustering and GLS algorithms to identify the optimum path for each cluster. This adaptation and optimization for the mTSP task necessitate a large amount of computing work and resources, which serves as a deterrent to attackers. (2) Mining incentives: Rewards for mining are dependent on the effectiveness of the optimized mTSP solution, which is confirmed by the SML and is intended to reward node integrity. Collaborating with mining nodes is discouraged to protect the integrity because the node requester has no incentive to accept an unqualified solution.

## Implementation details

### Dataset

The dataset that evaluated the experiments is called TSPLIB, as it includes a list of different instances, each of which has a number of locations represented in a two-dimensional coordinate system as described in^28^. Table 4 shows the scattering of locations, the number and the structure of some instances named ulyssess22, att48, eil101, and rd400.

**Table 4:** Data for some TSP-lib instances.

Name	ulyssess22	att48	eil101	rd400
Sample				
Number of locations	22	48	101	400
Data structure	Symmetric	Symmetric	Symmetric	Symmetric

### Performance evaluation

The performance of the proposed architecture in terms of the efficiency of the created clusters and the optimization algorithm utilized in solving the mTSP is evaluated by several criteria. These are (1) the summation of the squared error (SSE) for the clustering process; (2) the variance in the number of clusters; and (3) the full traveling distance.

### Sum of squared error

The Sum of Squared Error (SSE) is a popular criterion for clustering quality used to measure the variance within a cluster, as it is the sum of the squared distances between the locations and the closest centroids of the corresponding clusters, as in Eq. (10):

10 $$  SSE = {\text{min}}\mathop \sum \limits_{j = 1}^{K} \mathop \sum \limits_{i = 1}^{n} \left( {x_{i}^{\left( j \right)} - c_{j} } \right)^{2} ,j = 1, \ldots ,k $$

where *c*_*j*_ is the coordinate of cluster *j*, *x*_*i*_^*(j)*^ is the coordinate of location *i* in cluster *j*, *k* is the number of clusters, and the objective is to minimize this value.

### Elbow method

**Figure 7:**
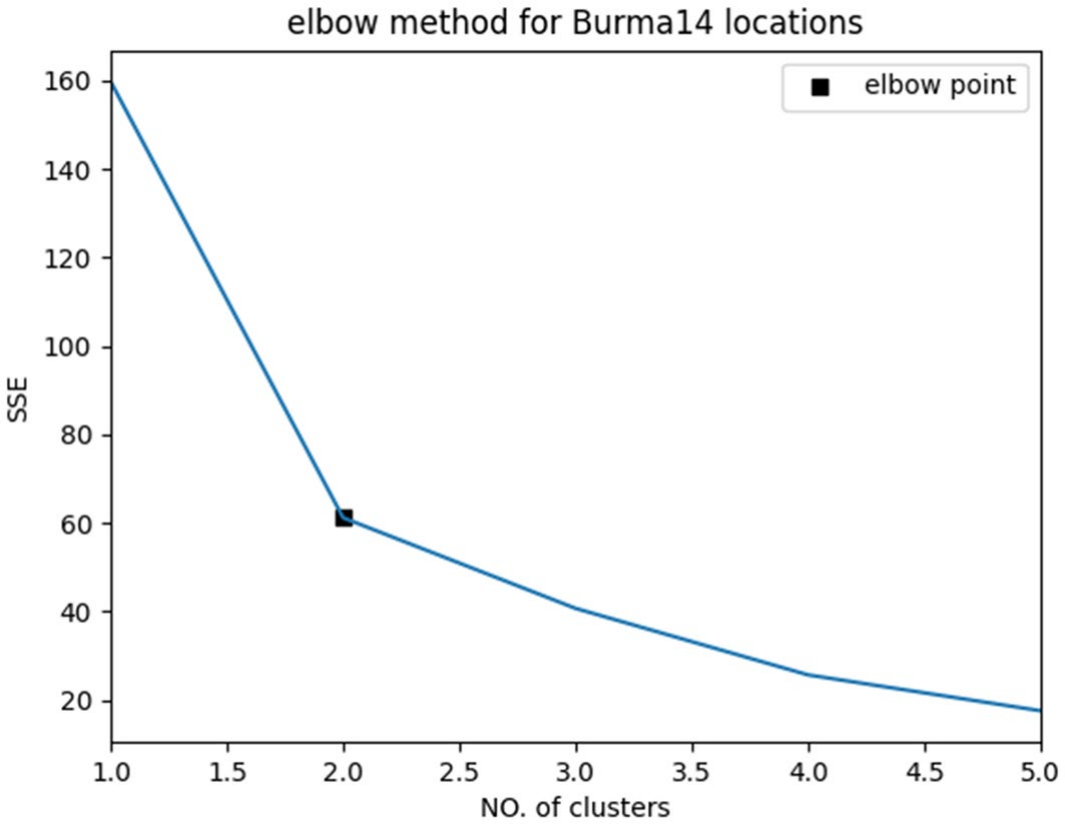
SSE versus No. of clusters plot representing elbow point (k = 2).

The k-means algorithm is preferred to use the elbow method to determine the best possible number of clusters within a range of values. It is a graphical method that requires drawing a line between the SSE and the k values to find the elbow point, after which SSE decreases in a linear direction. Figure 7 shows finding the best number of clusters for Burma14 locations using the elbow method.

### Total distance (cost)

The essential criterion of the mTSP solution is to reduce the traveled distance for each cluster and thus the total distance (D) as in Eq. (11):

11 $$  g\left( s \right) = min\mathop \sum \limits_{i,j \in s}^{n} d_{ij} ,d_{ij} = d_{ji} $$

where the goal function for solution *s* is *g*(*s*), *d*_*ji*_ is the distance between locations *i* and *j*, and *n* is the number of locations.

### Environment

Configuring the parameters to simulate the proposed architecture is shown in Table 5.

**Table 5:** Configured parameters to simulate the proposed architecture.

Environment	Parameters
System	Intel(R) Core (TM) i7-8550U CPU @ 1.80 GHz 1.99 GHz 8 GBRAM
Clustering	Python: Sklearn—Kmeans—Pandas—Matplotlib—Numpy
Path optimization	Python: Math—Or tools—Constraint_Solver—Routing_Enums_Pb2—Pywrapcp
Blockchain	Python: Flask—Requests—cryptography-rsa

## Experimental results

### Experiment 1: GLS preference over other TSP optimization methods

The ability of GLS to reach shorter distances, more efficient paths, and the best utilization of local search heuristics make it preferred over other TSP optimization techniques. We compared GLS with previous well-known meta-heuristics such as the genetic algorithm (GA)^29^, ant colony optimization (ACO)^30^, artificial bee colony (ABC)^31^, and monarchy metaheuristic (MN2)^32^ by testing it on various sizes of TSPLIB instances, the shortest distances, measured in kilometers, were obtained as shown in Table 6. Table 7 shows the enhancement percentages of GLS compared to the other algorithms. The overall percentage of improvement and distance reduction by applying GLS is around 0.17%.

**Table 6:** Comparison of total distance (cost) for different TSP instances using other proposed metaheuristics and GLS.

Instance	Meta-heuristics algorithms				
	GA	ACO	ABC	MN2	GLS
Eil51	441	450.59	563.75	630	**417**
Berlin52	7745	7548.99	9479.11	7703	**7525**
st70	707	696.05	1162.12	682.66	**663**
eil76	558	554.46	877.28	540	**530**
pr76	189,659	110,462	195,198.9	109,021	**108,160**
Kroa100	21,566	22,455.89	49,519.51	22,363	**21,351**
Eil101	696	678.04	1237.31	630	**624**

*The bold values indicate better GLS performance at shorter distances.

**Table 7:** The percentage difference between GLS versus other metaheuristics.

Instance	GA	ACO	ABC	MN2
Eil51	− 0.06	− 0.08	− 0.27	− 0.34
Berlin52	− 0.03	− 0.01	− 0.21	− 0.03
st70	− 0.07	− 0.05	− 0.43	− 0.03
eil76	− 0.06	− 0.05	− 0.4	− 0.02
pr76	− 0.76	− 0.03	− 0.45	− 0.01
Kroa100	− 0.02	− 0.05	− 0.57	− 0.05
Eil101	− 0.12	− 0.08	− 0.5	− 0.01
Average	− 0.16	− 0.05	− 0.4	− 0.07

*Negative values indicate the percentage improvement for GLS.

### Experiment 2: mTSP solutions optimization

Employing K-means for clustering and GLS improves the efficiency of mTSP solutions, resulting in more efficient paths and a lower overall distance traveled by the salesman. Table 8 shows the performance of our proposed work to solve the mTSP compared to PCI proposed in^33^ and AC2optGA proposed in^34^ in cost (distance) term for 7 instances from TSPLIB. The table header has eight main columns: instance name, number of instance locations (n), number of clusters (k) determined by the elbow method, SEE for the corresponding k, the starting point (depot), which is the first node in each instance, and the last three columns: the cost of the proposed algorithm, PCI, and AC2OptGA. The results show that the Kmean-GLS had an impact on reducing overall distances and improving the mTSP solutions. Table 9 shows the enhancement percentages for our proposed architecture compared to others in reducing the total distance (D) by an approximate percentage of 0.025%.

**Table 8:** Comparison of the performance of our proposed algorithm kmeans-GLS versus PCI and AC2OPTGA.

Name	n	k	SEE	Depot	k means-GLS	PCI	AC2OptGA
					Cost	Cost	Cost
eil51	51	3	51,629.69891	(37, 52)	**487**	492	–
eil76	76	2	50,715.93421	(22, 22)	**566**	586	–
rat99	99	3	629,171.5695	(6, 4)	**1621**	1647	–
pr226	226	5	15,715,407,062	(15,625, 1150)	**153,842**	156,015	161,084
pr299	299	5	2,176,604,589	(2156, 1639)	**76,015**	75,064	77,810
pr439	439	5	11,066,209,717	(7125, 11,300)	**144,224**	147,308	149,675
pr1002	1002	4	48,117,441,742	(1150, 4000)	**321,320**	329,128	351,371

*The bold values indicate the shorter total distances obtained with the proposed algorithm.

**Table 9:** The percentage difference between the proposed architecture kmeans-GLS versus PCI and AC2OptGA.

Instance	kmeans-GLS versus PCI	kmeans-GLS versus AC2OptGA
eil51	− 0.02	–
eil76	− 0.04	–
rat99	− 0.02	–
pr226	− 0.02	− 0.05
pr299	0.02	− 0.03
pr439	− 0.03	− 0.04
pr1002	− 0.03	− 0.09
Average	− 0.02	− 0.03

*Negative values indicate that the proposed architecture performs better.

### Experiment 3: Block generation time variation

**Figure 8:**
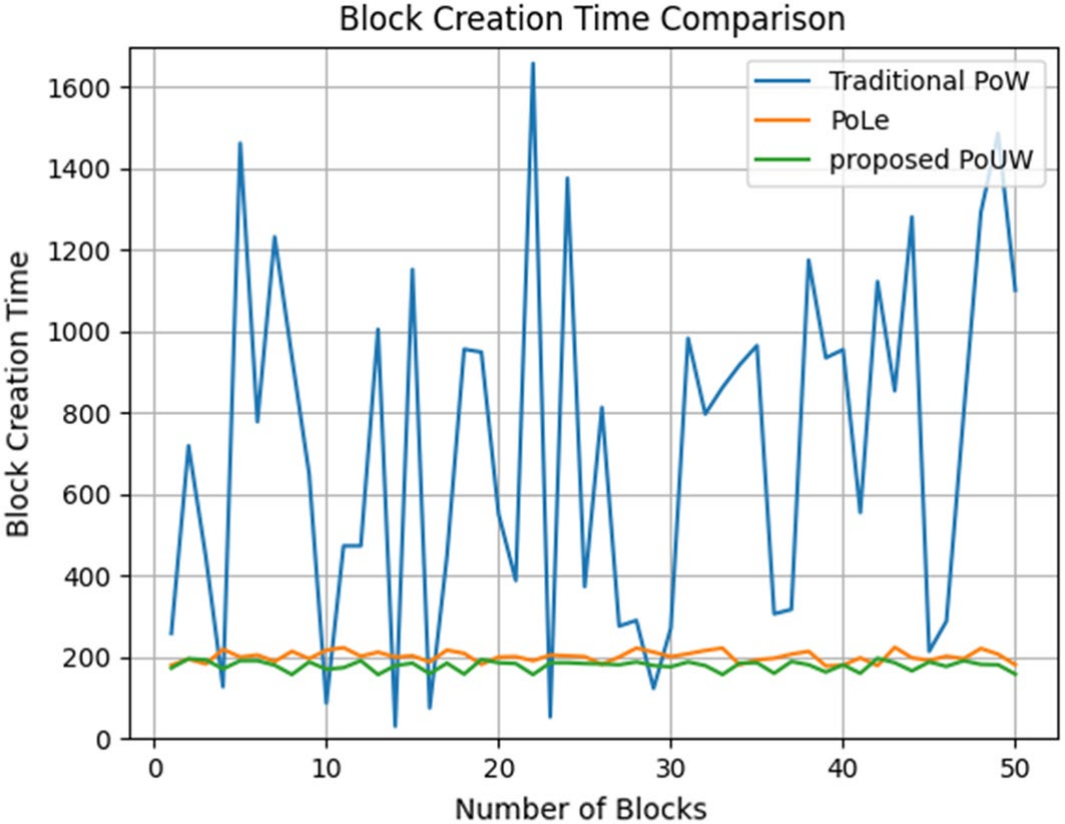
Block Generation Time of proposed PoUW, PoW, and PoLe.

The time it takes to create a block in a blockchain system is a vital factor in recording transactions and user experience in a distributed ledger system powered by blockchain technology, so we compared the proposed PoUW system with two other consensus algorithms: Traditional PoW with difficulty set to 23 and PoLe^14^ with accuracy model 0.8. In PoLe and the proposed PoUW, 5 data nodes and 3 consensus nodes were employed. We ran the systems to generate 50 blocks while recording the time taken for each block’s creation. Figure 8 shows how PoUW effectively overcomes the random variance of block generation time in PoW-based brute force computation and outperforms PoLe by a reduced variance of 120 to 180 s in contrast to the PoLe range of 200–250 s. That is, it decreased the block generation time (T) by 11.11%, which proves the ability of PoUW to improve the block generation time.

## Discussion

Our proposed PoUW significantly improved the blockchain by leveraging the capabilities of the PoW mechanism to improve mTSP. While the employment of k-means clustering and Guided Local Search (GLS) algorithm for miners efficiently improved mTSP, resulting in a reduction of distances compared to the previously proposed techniques, the innovation enhanced network performance by reducing block generation time as a result of miners employing a sequential technique to produce a valid block without compromising security, making it a promising solution for real-world applications.

## Conclusions and future work

Our proposed work focused on adapting the PoW mechanism to optimization issues and obtaining optimized solutions for mTSP, which enabled the exploitation of wasted energy while reducing the block generation time on the blockchain, so our work highlighted the exploitation of the PoW mechanism to resolve and improve real-world problems. Future research could focus on employing the resources of PoW on various optimization challenges with the potential to increase the security and control factors of the network.

## Data Availability

The datasets used and/or analyzed during the current study are available from the corresponding author on reasonable request.
